# A covalent peptide inhibitor of RGS4 identified in a focused one-bead, one compound library screen

**DOI:** 10.1186/1471-2210-9-9

**Published:** 2009-05-22

**Authors:** Rebecca A Roof, David L Roman, Samuel T Clements, Katarzyna Sobczyk-Kojiro, Levi L Blazer, Shodai Ota, Henry I Mosberg, Richard R Neubig

**Affiliations:** 1 Department of Pharmacology, University of Michigan,1301 MSRB III SPC 5632, 1150 W. Medical Center Dr, Ann Arbor, MI 48109, USA; 2 Department of Medicinal Chemistry, University of Michigan, 428 Church St, Ann Arbor, MI 48109, USA; 3 Current address: Molecular Neuropharmacology Section, National Institute of Neurological Disorders and Stroke, National Institutes of Health, 5625 Fishers Ln, Bethesda, MD 20852, USA; 4 Current address: College of Pharmacy, University of Iowa, 115 S. Grand Ave., Iowa City, IA 52242, USA

## Abstract

**Background:**

Regulators of G protein signaling (RGSs) accelerate GTP hydrolysis by Gα subunits and profoundly inhibit signaling by G protein-coupled receptors (GPCRs). The distinct expression patterns and pathophysiologic regulation of RGS proteins suggest that inhibitors may have therapeutic potential. We recently described a focused one-bead, one-compound (OBOC) library screen to identify peptide inhibitors of RGS4. Here we extend our observations to include another peptide with a different mechanism of action.

**Results:**

Peptide **5nd **(Tyr-Trp-c [Cys-Lys-Gly-Leu-Cys]-Lys-NH_2_, S-S) blocks the RGS4-Gα_o _interaction with an IC_50 _of 28 μM. It forms a covalent, dithiothreitol (DTT) sensitive adduct with a mass consistent with the incorporation of one peptide per RGS. Peptide **5nd **activity is abolished by either changing its disulfide bridge to a methylene dithioether bridge, which cannot form disulfide bridges to the RGS, or by removing all cysteines from the RGS protein. However, no single cysteine in RGS4 is completely necessary or sufficient for **5nd **activity.

**Conclusion:**

Though it has some RGS selectivity, **5nd **appears to be a partially random cysteine modifier. These data suggest that it inhibits RGS4 by forming disulfide bridges with the protein.

## Background

G Protein-Coupled Receptors (GPCRs) are a family of over 800 proteins that contain seven transmembrane regions [1]. When activated by agonists, such as hormones, neurotransmitters, drugs, or photons of light, GPCRs stimulate exchange of GTP for GDP on the Gα subunit of the heterotrimeric G protein, which then undergoes an activating conformational change that allows it and its associated βγ subunit to interact with effector proteins [2]. This can result in a number of downstream signaling events including changes in membrane potential, neurotransmitter or hormone release, gene transcription or other cellular events [1]. The Gα subunit inactivates itself by hydrolyzing GTP to GDP allowing reassociation with Gβγ and the receptor. Unlike GTPase accelerating proteins (GAPs) for small G-Proteins, regulators of G protein signaling (RGS) proteins bind to the activated Gα protein and stabilize the transition state for GTP hydrolysis without directly interacting with the nucleotide [3,4]. This accelerates GTP hydrolysis and inactivation of the G-protein, and inhibits cell responses to GPCR signaling.

We recently reported the screening of a focused, one-bead, one-compound (OBOC) peptide library for inhibitors of RGS4 [5]. This library was focused to include features known to be necessary for the activity of a lead peptide, **YJ34 **(Ac-Val-Lys-c [Cys-Thr-Gly-Ile-Cys]-Glu-NH_2_, S-S) which mimics the switch 1 region of Gα to bind to RGS4 and prevent its GAP activity [6]. This screen yielded two peptides, which have **YJ34**-like activities and, based on our models, bind the predicted **YJ34 **site on RGS4. Here we describe a third peptide from the library screen that has a different mechanism of action. The data presented here suggest that peptide **5nd **(Tyr-Trp-c [Cys-Lys-Gly-Leu-Cys]-Lys-NH_2_, S-S, peptide **7nd **in Roof et al, 2008), is a partially random cysteine modifier that inhibits RGS4 through disulfide bond formation between the peptide and the RGS.

## Results and discussion

**Figure 1:**
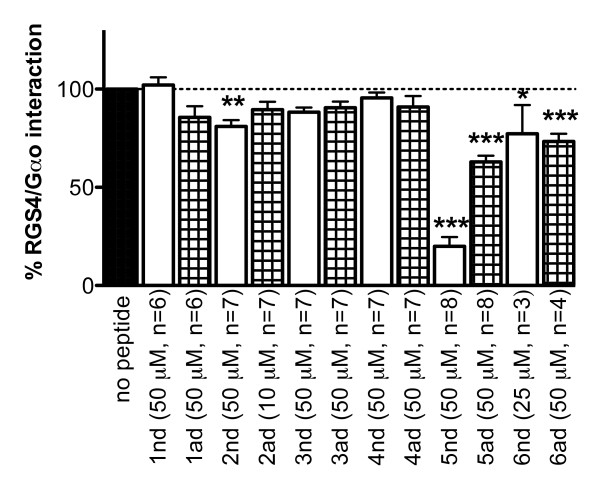
**Hit peptides in the FCPIA**. Hit peptides from Roof, et al. 2008 were tested at 50 μM unless otherwise indicated for ability to disrupt the RGS4-Gα_o _interaction as described using 25 nM Gα o-Alexa Fluor-532 and 5 nM wild type RGS4-biotin (mean ± S.E.M., n ≥ 3) *p < 0.05, **p < 0.01, ***p < 0.001 compared to no peptide.

We recently described a bead-based screen in which 2.5 × 10^6 ^different peptide sequences were prepared as a OBOC library [5]. Fluorescently labeled RGS4 was used to probe the library for peptides that bind RGS4 [5]. A total of 14 peptide sequences were obtained and, in our first report, one peptide sequence (in two chemical forms) was found to inhibit RGS4-stimulated GTPase activity. Since the majority of those hit peptides could bind tightly to the RGS without disrupting GAP activity, they were also tested in the flow cytometry protein interaction assay (FCPIA) (Figure 1), which measures the interaction between RGS4 and Gα_o_. In this method we used a Luminex flow cytometer to detect the binding of Alexa Fluor 532-labeled Gα_o _to biotinylated RGS4 on avidin beads in the presence of AMF (AlCl_3_, MgCl_2_, NaF and GDP) to put the Gα subunit into a transition state-like conformation [7]. Peptides were tested at 50 μM except where limited by solubility (Table 1). Fourteen peptide sequences obtained from the OBOC screen, in both acetylated, disulfide bridged (ad) or free N-terminal disulfide bridged (nd) versions (or 28 peptides total), were tested and 5 showed statistically significant inhibition of the RGS4-Gα_o _interaction in the FCPIA assay (**2nd**, **5nd**, **5ad**, **6nd **and **6ad**, Figure 1, Table 1). Peptide **5 **was the most active (80 ± 5 and 37 ± 3 percent inhibition for **5nd **and **5ad**, respectively) (Figure 1).

**Table 1:** Hit peptide names, sequences and physical properties

Name	Sequence	cyclization	Buffer Solubility (μM)	ALogP	% Inhibition of RGS4-Gα_o_
YJ34	Ac-VKCTGICE-NH_2_	S-S	80	-2.58	-17 ± 4 (40 μM)

1nd	YNCQGECK-NH_2_	S-S	≥ 420	-2.8	2 ± 4

1ad	Ac-YNCQGECK-NH_2_	S-S	123	-2.37	14 ± 6

**2nd**	**GTCFGTCW-NH_2_**	**S-S**	**464**	**-0.37**	19 ± 3**

2ad	Ac-GTCFGTCW-NH_2_	S-S	30	0.08	10 ± 4 (10 μM)

3nd	LVCKGYCQ-NH_2_	S-S	≥ 470	-0.37	12 ± 2

3ad	Ac-LVCKGYCQ-NH_2_	S-S	427	0.17	10 ± 3

4nd	KVCMGGCT-NH_2_	S-S	≥ 470	-2.02	4 ± 3

4ad	Ac-KVCMGGCT-NH_2_	S-S	459	-2.09	9 ± 6

5nl	YWCKGLCK-NH_2_	Linear	465	1.46	-1 ± 10

5al	Ac-YWCKGLCK-NH_2_	Linear	460	1.66	-2 ± 4

5nd*	**YWCKGLCK-NH_2_**	**S-S**	**463**	**0.48**	80 ± 5***

5ad*	**Ac-YWCKGLCK-NH_2_**	**S-S**	**464**	**1.04**	37 ± 3***

5nm	YWCKGLCK-NH_2_	S-me-S	≥ 470	0.53	3 ± 0.1 (100 μM)

6nd*	**KHCYGFCY-NH_2_**	**S-S**	**Low**	**0.94**	23 ± 15 (25 μM) *

6ad*	**Ac-KHCYGFCY-NH_2_**	**S-S**	**421**	**1.24**	27 ± 4***

**Figure 2:**
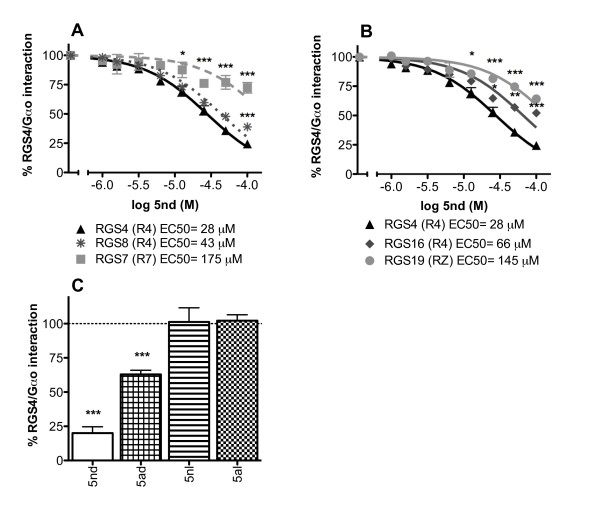
**Characterization of hit 5**. A) and B) Dose response curves for **5nd **in the FCPIA with different RGS proteins (RGS Family), (mean ± S.E.M., n = 3) *p < 0.05, **p < 0.01, ***p < 0.001 compared to RGS4 at the same **5nd **concentration. C) Peptides were tested at 50 μM as in Figure 1, (mean ± S.E.M., n ≥ 3) *p < 0.05, **p < 0.01, ***p < 0.001 compared to no peptide.

Solubility in buffer was determined experimentally by HPLC. ALogP calculations are from , [9]. Percent inhibition values (50 μM unless otherwise stated) are from Figure 1, 2C, or 3A (mean ± S.E.M., n ≥ 3) *p < 0.05, **p < 0.01, ***p < 0.001 compared to no peptide. Peptides with significant inhibition in the FCPIA are in bold. *Peptides 5 and 6 were previously reported as peptides 7 and 14, respectively in Roof et al 2008.

**5nd **inhibited Gα_o _binding to a number of different RGS proteins (Figure 2). The IC_50 _values are 28 μM, 43 μM, 66 μM, ~145 μM and ~175 μM for RGS4, RGS8, RGS16, RGS19 and RGS7, respectively (logIC_50 _of -4.56 ± 0.02, -4.36 ± 0.02, -4.18 ± 0.03, ~-3.84 ± 0.05 and ~-3.76 ± 0.08). The most potent effects were on RGS4, RGS8 and RGS16, which all belong to the same RGS family (R4) and share the greatest sequence homology. RGS19 and RGS7 are in the RZ and R7 families, respectively and show less inhibition by **5nd**. Linear as well as bridged versions of peptide **5 **were tested in the FCPIA since cyclization yields would most likely have been less then 100% in the synthesis of the library leaving some linear peptides present on each bead. The free N-terminal linear (nl) and acetylated linear (al) peptides were found to be inactive (-1 ± 10 and -2 ± 4 percent inhibition for **5nl **and **5al**, respectively) (Figure 2C). This may be due to lack of structural rigidity necessary for the correct interaction of the peptide with the RGS protein or a role for chemical reactivity of the disulfide.

**Figure 3:**
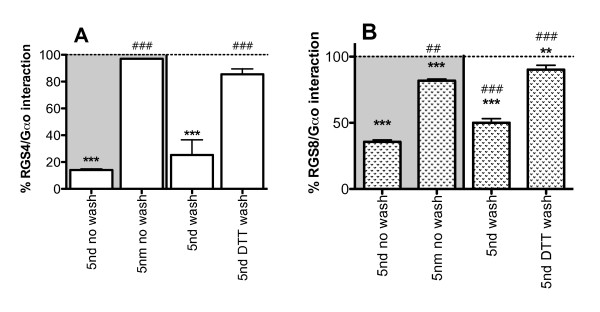
**Irreversibility experiments**. RGS-biotin on avidin beads was treated with **5nd **(100 μM) or **5nm **(100 μM) and then washed in buffer (with or without 1 mM DTT) or not washed and put in the FCPIA using RGS4 (A) or RGS8 (B) (mean ± S.E.M., n ≥ 2) *p < 0.05, **p < 0.01, ***p < 0.001 compared to no peptide (100%); #p < 0.05, ##p < 0.01, ###p < 0.001 compared to **5nd **no wash.

We next wanted to determine the mechanism of action of peptide **5**. A small molecule inhibitor of RGS4, methyl N-[(4-chlorophenyl)sulfonyl]-4-nitrobenzenesulfinimidoate (CCG-4986) was identified in a FCPIA screen and found to interact with RGS4 through cysteine modification ([7,8], David Roman, in preparation). We wanted to determine if peptide **5nd **acts in a similar manner. When biotin-RGS4 on avidin beads was treated with **5nd**, followed by washing of the beads, the inhibition of RGS4-Gα_o _interactions was not reversed (75 ± 11 percent inhibition remained). Inclusion of dithiothreitol (DTT) in the wash buffer significantly decreased **5nd **activity (15 ± 4 percent inhibition) (Figure 3A). These data suggest that the peptide may bind irreversibly through a disulfide bridge. To further investigate this possibility, a free N-terminal, methylene dithioether bridged peptide, **5nm **(Table 1), was synthesized and found to be inactive (3 ± 0.1 percent inhibition) (Figure 3A). Since the methylene dithioether bridged peptide would be incapable of forming a disulfide bond with RGS4, this result supports the hypothesis that **5nd **forms a functionally critical disulfide bridge with RGS4. Although, it is also possible that the structural change from the increased bridge length is responsible for the loss of activity of **5nm **compared to **5nd**. A similar pattern was seen with RGS8; the loss of activity of **5nd **on RGS8 was much greater with washing if DTT was included in the buffer and **5nm **had only a small effect on RGS8 activity (64 ± 2, 50 ± 3, 10 ± 3, and 18 ± 1 percent inhibition for **5nd **no wash, **5nd **wash, **5nd **DTT wash and **5nm **respectively) (Figure 3B).

**Figure 4:**
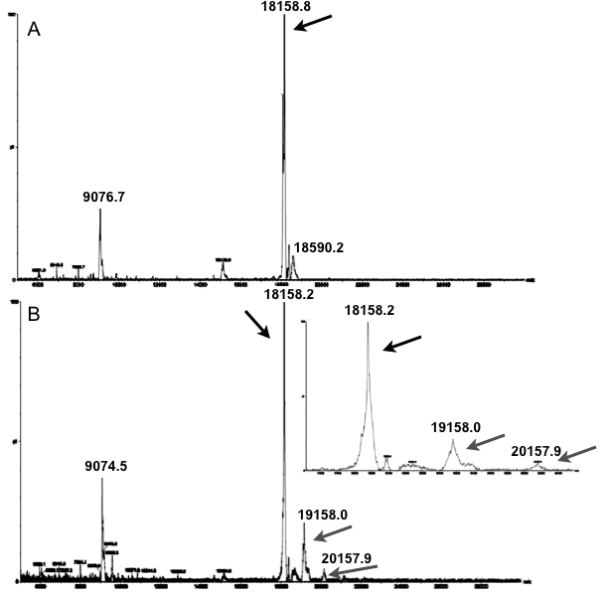
**MS analysis of RGS4 with and without 5nd pretreatment**. RGS4Δ51N was cleaved from the MBP-His_6 _construct and subject to MALDI MS after pretreatment with DMSO (A) or **5nd **(250 μM) (B). Both spectra show the region between 4,000 and 30,000 m/z and have the peak at 18158.8 or 18158.2 m/z for A and B, respectively, which corresponds to the cleaved RGS4Δ51N protein (black arrows). The spectrum in B also has a peak at 19158 m/z that corresponds to a peptide adduct (grey arrows). There is a very small peak at 20157.9 that may correspond to 2 peptides per RGS (grey arrows). The inset is a blow-up of the region between 17500 and 20500 m/z.

To directly test for the formation of a covalent adduct between **5nd **and RGS4, we performed mass spectrometry (MS) analysis. The RGS4Δ51N protein, following TEV-protease cleavage from the MBP-His_6 _construct, was treated with **5nd **at a 50:1 molar ratio. An adduct to the protein that is consistent with the mass of **5nd **binding through a disulfide bridge was observed by MS (Figure 4, mass shift observed: Δ999.2 ± 1; expected: Δ998.5). No such shift was observed with DMSO-treated RGS4Δ51N. There is also a small peak that may represent two peptides per RGS (observed: Δ1999.1 ± 2; expected: Δ1997.0).

**Figure 5:**
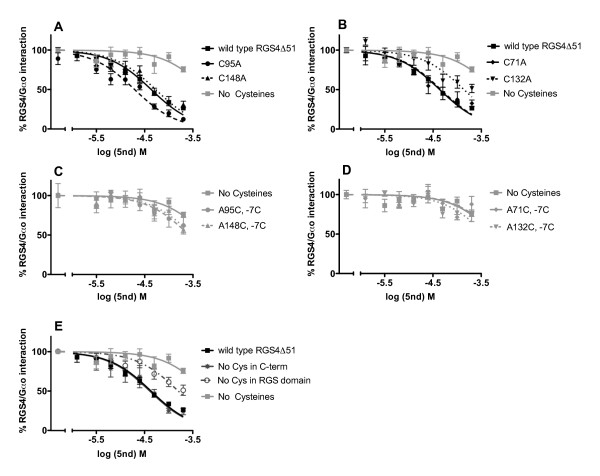
**Dose curves of 5nd on various RGS4 cysteine mutants**. Various concentrations of **5nd **were tested for disruption of the RGS4-Gα_o _interaction for C95A (5 nM), or C148A (5nM) (A), C71A (5nM) or C148A (5nM) (B), A95C in the -7C (5nM), or A132C in the -7C (5nM) (C), A71C in the -7C (5nM), or A148C in the -7C (5nM) (D) or the mutant with no cysteines in the domain (20 nM) or the C-terminus (20 nM) (E) in the FCPIA (mean ± S.E.M., n ≥ 3). See Table 2 for statistics.

Since **5nd **forms an irreversible, DTT-sensitive bond with RGS, it was suspected that it binds covalently to a cysteine in the protein through a disulfide bridge. Indeed, removal of all 7 cysteines (termed the -7C mutant) from RGS4 greatly diminished **5nd **activity (logIC_50 _of -3.24 ± 0.14 for -7C vs. -4.36 ± 0.02 for wild type) (Figure 5, Table 2). Removal of cysteines from the C-terminus of RGS4 had no effect on the potency of **5nd **(logIC_50 _-4.5 ± 0.2) while removal of all 4 cysteines from the RGS domain did reduce the potency of compound by 3.6-fold (logIC_50 _-3.8 ± 0.2) (Figure 5E, Table 2). These results suggest a complex mechanism involving cysteines in both the C-terminus and RGS domain based on the discrepancy in 5nd potency on the -7C mutant and the protein with no cys in the RGS domain.

**Table 2:** Mutant RGS4 Gα_o _binding affinities and 5nd logIC_50_s.

**Mutant**	**Gα_o _affinity (nM)**	**5nd logIC_50 _(M)**
wild type	11.6 ± 1.4	-4.36 ± 0.03###

C95A	8.17 ± 1.5	-4.71 ± 0.05###

C148A	6.84 ± 1	-4.27 ± 0.05#

C71A	12.2 ± 1.8	-4.34 ± 0.06##

C132A	7.9 ± 3.1	-3.86 ± 0.07

-7C	8.3 ± 1	-3.24 ± 0.14***

A95C, -7C	12 ± 2	-3.58 ± 0.09**

A148C, -7C	5.7 ± 0.2	-3.51 ± 0.09**

A71C, -7C	9.2 ± 2	-3.20 ± 0.13**

A132C, -7C	12.5 ± 1	-3.41 ± 0.09**

no Cys in RGS domain	3.9 ± 0.8	-3.80 ± 0.06*

no Cys in C-terminus	5.0 ± 0.7	-4.39 ± 0.07##

Gα_o _affinities were calculated in the FCPIA as described and logIC_50 _data is from Figure 5. (mean ± S.E.M., n ≥ 3) *p < 0.05, **p < 0.01, ***p < 0.001 compared to wild type RGS4Δ51N; #p < 0.05, ##p < 0.01, ###p < 0.001 compared to -7C RGS4Δ51N.

**Figure 6:**
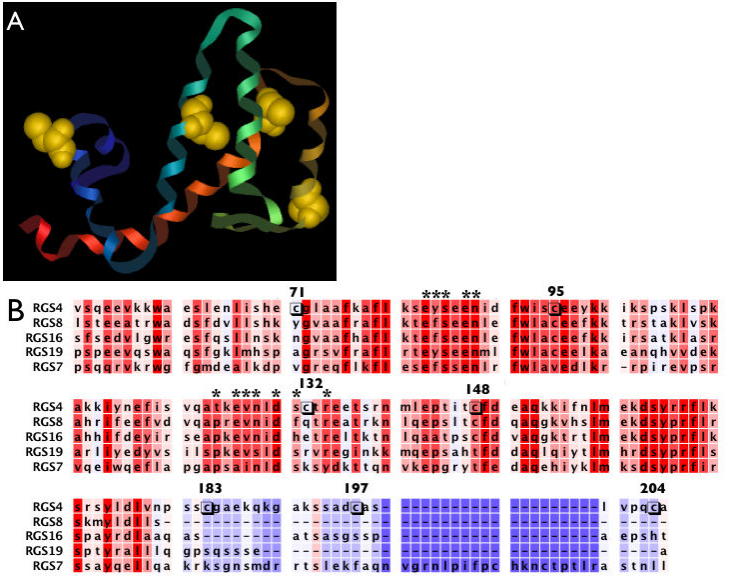
**RGS alignment**. A) The structure of the RGS4 domain [3] with the cysteines highlighted. B) An alignment of the RGS proteins used in Figure 3 compared to the RGS4Δ51N construct. The RGS proteins are listed in order of inhibition by **5nd**. The red residues are conserved and the blue are not. The stared residues are important for Gα interactions and the cysteines in RGS4 are boxed.

To further explore the role of cysteine residues, the RGS proteins tested in Figure 2 were aligned with RGS4 (Figure 6) to identify shared cysteines. Based on the conservation of Cys95 and Cys148 in RGS4 RGS8 and RGS16, which are all inhibited by **5nd**, it was hypothesized that those cysteines may be involved in the peptide's activity. However, removing those cysteines individually (i.e. in the C95A and C148A mutant RGS4) did not diminish **5nd **activity (logIC_50 _-4.71 ± 0.0.5 and -4.27 ± 0.05 for C95A and C148A, respectively) (Figure 5A, Table 2). Because all the mutants used in this manuscript bound Gα_o _in an AMF-dependent manner with reasonable affinities compared to wild type (Table 2), it is reasonable to assume they are folded properly. With the assumption that **5nd **would have to bind within the RGS domain to inhibit Gα_o _binding, C71A and C132A mutations were also tested. The C71A mutation did not affect **5nd **activity (logIC_50 _-4.34 ± 0.06). The C132A mutation did decrease **5nd **potency, but only partially (logIC_50 _-3.86 ± 0.07) (Figure 5B, Table 2). Interestingly, C132 is near the Gα binding site, which is also the suggested YJ34 binding site. It is tempting to speculate that one of the multiple sites of action of **5nd **is adjacent to the Gα binding site (similar to that of **YJ34)**, however, the A132C "add back" mutant discussed below again suggests a complex scenario.

The previous experiments tested which cysteines are necessary for inhibition by **5nd**. In an alternative approach, we added cysteines back to the -7C mutant to determine which might be sufficient for **5nd **activity. Surprisingly, no single A to C mutation within the RGS domain of the -7C mutant even partially restored **5nd **activity; not even the A132C mutant (Figure 5C, Figure 5D, Table 2). This suggests that **5nd **inhibits RGS4/Gα_o _interactions by binding to multiple cysteines – probably in both the RGS domain and the C-terminus. Furthermore, Cys132 is involved in the actions but this is clearly not sufficient to explain them. Thus it is concluded that **5nd **is at least partially non-selective in its cysteine modification. These data also suggest RGS4 is more sensitive to covalent redox manipulations than are the other RGS proteins tested.

In summary, peptide **5nd **binds covalently through disulfide bridges with cysteines in the protein and it raises some interesting points regarding the previously reported focused OBOC screen [5]. First, it is interesting that although the library was focused to include features necessary for **YJ34 **activity, peptide **5nd **was isolated that clearly works through a different mechanism. This was unexpected since the library was biased towards peptides that would have the same mechanism as the lead compound. However, this bias is by no means a guarantee. Indeed, there is no way to know whether a peptide like hit **2**, (which has **YJ34**-like activities and is modeled to bind the putative **YJ34 **site on RGS4, [5]) would have been found from a completely random library.

Another interesting observation is that RGS4 is preferentially inhibited by the cysteine modifier peptide over other RGS proteins. This could be because the peptide binds selectively to a pocket on RGS4, or because RGS4 is particularly susceptible to cysteine modification. This latter possibility is supported by the observation that a small molecule inhibitor of RGS4, CCG-4986, that was identified in an FCPIA screen appears to inhibit RGS4 through covalent modification of cysteines while having no activity against RGS8 ([7,8], David Roman, in preparation). Also, RGS4 is more sensitive to inhibition with N-ethylmaleimide, (a cysteine modifier) than RGS8 (David Roman, personal communication). Unlike **5nd**, CCG-4986 appears to selectively modify 1 or 2 cysteines in the RGS4 ([7,8], David Roman, in preparation). This increased cysteine selectivity may be why CCG-4986 has more RGS selectivity than **5nd**. However, since all peptides in our library have a disulfide bond, it is not clear why **5nd **(and **5ad**) would be so much more potent at covalently modifying RGS4. Perhaps the cysteine reactive peptide also has some affinity for a specific pocket on the RGS.

A third observation is that there may be a correlation between peptide logP and activity in the FCPIA (Table 1). The order of activity in the FCPIA is hit **5**>hit **6**>hit **2**> others [9], while the order of hydrophobicity is hit **6**>hit **5**>hit **3**>hit **2**> others. Given the considerable error in computational logP calculations [10], it is reasonable to suggest at least qualitatively that the most hydrophobic peptides have the greatest activity. This also tells us something about RGS4. These data suggest that RGS4 binding sites, including unidentified binding sites, have hydrophobic surfaces and investigators should be mindful of this when choosing libraries for future screens.

## Conclusion

We report the identification and characterization of a peptide RGS4 inhibitor with a novel mechanism involving cysteine-modification. Although our focused OBOC screen did not improve on the potency of existing RGS4 inhibitors with, these data illustrate points that should result in improved screens in the future.

## Methods

### Materials

Fmoc-protected amino acids and Rink amide resin were purchased from Advanced ChemTech. Peptide synthesis grade chemicals were purchased from Applied Biosystems. Avidin coated microspheres were purchased from Luminex.

### Protein Expression, Purification and Labeling

Proteins were expressed, purified and labeled as described previously [5]. The mutagenesis was done using the "QuickChange Multi Site-Directed Mutagenesis Kit" from Stratagene according to the manufacturer's protocol. The construct of RGS4 used in these studies is a maltose binding protein (MBP-) fusion protein of the RGS4 sequence from which the amphipathic N-terminal helix has been removed (i.e. MBP-RGS4Δ51N). For simplicity in the text, this will be referred to as RGS4. All other RGS proteins were human sequences and were used as Mpb fusions: RGS7 RGS domain (aa: 305–453), RGS8 full length, RGS16 full length, and RGS19ΔC11 (amino acids 1–209). To cleave RGS4Δ51N from the MBP-His_6 _construct for MS analysis, MBP-His_6_-RGS4Δ51N was treated with 15% MBP-His_6_-TEV protease S219V [11] for 22 hours at 4°C and purified over a Ni-NTA column.

### Peptide Synthesis

Soluble peptides were synthesized on Rink resin, cleaved and cyclized as described previously [6]. Peptide purity (at least 95%) and solubility were verified by high-performance liquid chromatography and correct mass was verified by MS analysis [6]. ALogP calculations are from [9]. Peptides were prepared as 5 mM stocks in DMSO.

### FCPIA (Flow Cytometry Protein Interaction Assay)

FCPIA was performed as previously published [7].

### Mass Spectrometry

Total mass MS of 5 μM RGS4Δ51N was analyzed on a MicroMX MALDI MS instrument in positive ion mode with an accuracy of ± 0.1% after pretreatment with DMSO or 250 μM **5nd**. The sample was run over a desalting column prior to MS analysis. MS analysis was performed at the Protein Structure Facility at the University of Michigan.

### Statistical Analysis

Data are expressed as mean ± S.E.M (or ± S.D for n = 2) and analyzed by one-way ANOVA. Dose curves were constrained with maximum and minimum at 100% and 0%, respectively. Statistics for Figure 5 are included in Table 2 instead of on the graph for simplicity. Significance is indicated as follows: *p < 0.05, **p < 0.01, ***p < 0.001. Analysis was performed using Graphpad Prism.

## Abbreviations

AMF: AlCl_3_, MgCl_2_, NaF and GDP; ANOVA: analysis of variance; DMSO: dimethylsulfoxide; DTT: dithiothreitol; GAP: GTPase accelerating protein; GPCR: G protein-coupled receptor; GTP: guanosine triphosphate; FCPIA: flow cytometry protein interaction assay; OBOC: one-bead, one-compound; RGS: regulator of G protein signaling; SAR: structure activity relationship.

## Authors' contributions

RR, HM, and RN conceived the project; RR synthesized the library and ran the initial screen. She also synthesized some of the peptides, prepared some of the proteins, designed and performed the FCPIA experiments except Figure 2 which was performed by STC. RR also analyzed the data, prepared samples for MS, and drafted the manuscript. DR taught RR the FCPIA assay, designed and carried out most of the DNA mutagenesis, prepared and labeled many of the proteins used, and determined Gα_o _affinities for most of the RGS4Δ51N mutants. KS provided assistance and advice with the peptide synthesis. LB and SO prepared some of the proteins used. RR, DR, LB, and SO all did some of the protein labeling. HM and RN oversaw the project. All authors read and approved the manuscript.
